# Use of gene expression profiling to identify candidate genes for pretherapeutic patient classification in acute appendicitis

**DOI:** 10.1093/bjsopen/zraa045

**Published:** 2021-01-09

**Authors:** N Kiss, M Minderjahn, J Reismann, J Svensson, T Wester, K Hauptmann, M Schad, J Kallarackal, H von Bernuth, M Reismann

**Affiliations:** 1 Department of Paediatric Surgery, Charité – Universitätsmedizin Berlin, Berlin, Germany; 2 Department of Paediatric Surgery, Astrid Lindgren Children's Hospital, Karolinska University Hospital, Stockholm, Sweden; 3 Institute of Pathology, Charité – Universitätsmedizin Berlin, Berlin, Germany; 4 OakLabs, Hennigsdorf, Germany; 5 Department of Paediatric Pulmonology and Immunology, Charité – Universitätsmedizin Berlin, Berlin, Germany

## Abstract

**Background:**

Phlegmonous and gangrenous appendicitis represent independent pathophysiological entities with different clinical courses ranging from spontaneous resolution to septic disease. However, reliable predictive methods for these clinical phenotypes have not yet been established. In an attempt to provide pathophysiological insights into the matter, a genomewide gene expression analysis was undertaken in patients with acute appendicitis.

**Methods:**

Peripheral blood mononuclear cells were isolated and, after histological confirmation of PA or GA, analysed for genomewide gene expression profiling using RNA microarray technology and subsequent pathway analysis.

**Results:**

Samples from 29 patients aged 7–17 years were included. Genomewide gene expression analysis was performed on 13 samples of phlegmonous and 16 of gangrenous appendicitis. From a total of 56 666 genes, 3594 were significantly differently expressed. Distinct interaction between T and B cells in the phlegmonous appendicitis group was suggested by overexpression of T cell receptor α and β subunits, CD2, CD3, MHC II, CD40L, and the B cell markers CD72 and CD79, indicating an antiviral mechanism. In the gangrenous appendicitis group, expression of genes delineating antibacterial mechanisms was found.

**Conclusion:**

These results provide evidence for different and independent gene expression in phlegmonous and gangrenous appendicitis in general, but also suggest distinct immunological patterns for the respective entities. In particular, the findings are compatible with previous evidence of spontaneous resolution in phlegmonous and progressive disease in gangrenous appendicitis.

## Teaser

Reliable distinction of phlegmonous and gangrenous appendicitis is not possible by current means. The aim of this study was to test the applicability of gene expression data including a pathway analysis for differentiation of the entities.

## Introduction

Surgeons know how to treat appendicitis: either surgically with appendicectomy and supporting measures like abscess drainage, or conservatively without operation^1^. Nevertheless, the optimal treatment depends on the stage or type of inflammation of the vermiform appendix.

The presence of necrosis seems to be the determining factor: necrotizing gangrenous appendicitis (GA) is associated with a complicated clinical course including increased rates of peritonitis and abscess formation, even if perforation is not present, whereas non-necrotizing, phlegmonous appendicitis (PA) usually seems to be self-limiting^1–3^. Further insights into epidemiological aspects have shown that perforated and non-perforated appendicitis are characterized by independent frequencies within defined populations^4^. Histologically, perforated GA, referred to clinically as complicated appendicitis, seems to evolve much faster than previously thought, with increased risk of abscess formation up to septic disease, and can rarely be prevented. In contrast, uncomplicated, non-perforated inflammation appears to be self-limiting, with spontaneously decreasing inflammatory values in a large proportion of those affected^5^. These epidemiological data are supported by evidence from an RCT^5^ comparing treatment results for patients who had been diagnosed with uncomplicated appendicitis, and were treated conservatively with or without antibiotics. Rates of treatment failure were comparable in the two arms (79.3 *versus* 76.6 per cent). Further support for the hypothesis of independent inflammatory entities is provided by immunological evidence. PA and GA differ significantly in terms of distinct underlying immunological patterns; there are primarily Th2-helper cell-dependent mechanisms in those with PA and Th1/Th17-dependent mechanisms in patients with gangrenous inflammation^6^^,^^7^.

As the inflammatory entity seemingly determines the clinical course, these types of appendicitis require different therapeutic courses of action. Therefore, decisive diagnostics are mandatory to reliably differentiate complicated from uncomplicated appendicitis.

Clinical investigation and laboratory values are basic methods used in the diagnosis of acute appendicitis^8^. In an analysis of the use of routine laboratory values (full blood count and C-reactive protein) for pretherapeutic prediction of PA and GA, the parameters tested did not provide a decisive distinction between the two, although steady pathophysiological differences between the histopathological entities were demonstrated^9^.

Currently, imaging techniques such as CT seem to provide good capacity for differentiation between complicated and uncomplicated appendicitis^10^. In a study^11^ focused on the value of radiation-free sonography for differentiation of the inflammatory types in children, abdominal ultrasonography proved to be a valuable tool for the diagnosis of appendicitis, but was limited in terms of differentiation of the two entities. Application of artificial intelligence and methods of machine learning to the laboratory parameters and sonographically measured appendiceal diameters led to substantially improved capacity for the discrimination of PA and GA, and thereby computer algorithm-based biomarker signatures were established^12^.

This comprehensive prospective whole-genome gene expression study essentially encompasses two objectives. First, the aim was to investigate the general extent of gene expression differences in children with PA and GA. Second, the entities were examined at a basic level of gene expression that would allow description of specific pathophysiological and, particularly, immunological differences in terms of pathways. Interesting differences between PA and GA have already been described at the genomic level^13^. The present description of dynamic gene expression level with subsequent pathway analysis for differential diagnosis of acute appendicitis is a novel approach.

## Methods

This was a single-centre, prospective study of patients aged 7–17 years who had undergone surgery for acute appendicitis at the Department of Paediatric Surgery of Charité – Universitätsmedizin Berlin, between April and August 2019. The specific age group in this paediatric surgical population was determined as a necessary level of ability to consent and reading comprehension was required in order to be able to give informed consent to participate in the study. The study was approved for the limited number of patients (30 + expected exclusion rate of 10 per cent) by the institutional ethics committee (reference number ES2/130/16), with the perspective of a subsequent sample size calculation for a larger study.

Inclusion criteria for the study were: patients aged 7–17 years with a sonographically suspected diagnosis of acute appendicitis and scheduled for appendicectomy^5^. Patients and parents were informed by means of appropriate age-adapted information sheets (children aged 7–14 and 15–17 years; separate leaflets for parents) to obtain informed consent. Patients were specifically asked to report the onset of abdominal pain (± 30 min). The interval between the start of symptoms and blood sample collection was calculated accordingly.

Exclusion criteria were: missing consent; concomitant disease; previous or present specific treatment for acute appendicitis (conservative or surgical); antibiotic treatment within the past 2 weeks; interval between blood sample collection and isolation of peripheral blood mononuclear cells (PBMCs) more than 1 h; insufficient RNA quality (RNA integrity number (RIN) below 7); and appendicectomy not performed (no histopathological specimen).

In strict accordance with the ethics committee’s approval, blood samples (at least 5 ml) were drawn during routine medical laboratory investigations in the paediatric emergency department. The isolation of PBMCs was performed within 1 h after blood sampling. The blood from each individual was suspended in phosphate-buffered saline (PBS) at a 1 : 1 ratio. Density gradient centrifugation was performed using Ficoll^®^ PM400 (GE Healthcare, Pittsburgh, Pennsylvania, USA) at room temperature for 30 min at 400*g*. After isolation of the monocyte layer, the cells were resuspended in PBS and centrifuged twice for 5 min at 400*g*. After final resuspension in 1 ml PBS, centrifugation and removal of the supernatant, the native cells were frozen at –20°C using a sample freezing container (Mr. Frosty™, Thermo Fisher Scientific Inc., Waltham, Massachusetts, USA) and finally stored at –80°C in liquid nitrogen.

### Histopathological examination

**Fig. 1:**
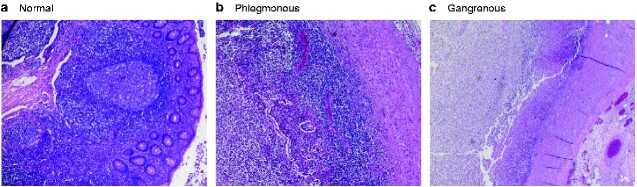
Appendiceal histology **a** Uninflamed appendix with normal lymphofollicular structures, **b** phlegmonous appendicitis with oedema and diffuse transmural granulocytic infiltration, and **c** gangrenous appendicitis with almost complete wall necrosis and fibrinous–purulent inflammation of serosa and adjacent fatty tissue

After appendicectomy, the resected appendixes were classified histopathologically according to Carr^2^. PA is characterized by transmural infiltration of the appendix by neutrophilic granulocytes, serositis, microabscesses, and oedema, without gangrene or perforation. GA is especially characterized by ischaemic areas in the appendix with transmural myonecrosis. Perforated appendicitis is defined by gangrenous alterations with a transmural defect of the appendix wall. *Fig. 1* shows the major histopathological features for group assignment (phlegmonous *versus* gangrenous) in comparison to normal appendix.

After the primary histopathological examination, a second blinded evaluation was undertaken by a specialized paediatric pathologist.

### Laboratory workflow

Total RNA was isolated from PBMCs using a NucleoSpin™ RNA Plus kit (Macherey-Nagel, Germany). RNA quality control was achieved using a 2100 Bioanalyzer (Agilent Technologies, Santa Clara, CA, USA) and the RNA 6000 Pico Kit for total RNA samples. For quantitative control, photometric measurement was performed using a NanoDrop™ 2000 spectrophotometer (Thermo Fisher Scientific Inc., Waltham, Massachusetts, USA). The quality of each sample was evaluated based on the Bioanalyzer RIN. Samples with a RIN above 7 fulfilled quality control and were subjected to labelling.

For labelling, a Low Input QuickAmp Labeling Kit (Agilent Technologies, Santa Clara, CA, USA) was used to generate fluorescent complementary RNA (cRNA). A random primer–oligo-dT primer mixture was used for first-strand synthesis. After second-strand synthesis, *in vitro* transcription for synthesis of cRNA labelled with cyanine 3-CTP was performed.

Some 600 ng of each Cy3-labelled cRNA was hybridized at 65°C for 17 h on an OakLabs 8x60K ArrayXS Human Agilent microarray (design ID 79407) containing probes for 56 666 genes, using an Agilent Gene Expression Hybridization Kit (Agilent Technologies, Santa Clara, CA, USA), followed by microarray wash and scanning on a SureScan Microarray Scanner (Agilent Technologies, Santa Clara, CA, USA) at a resolution of 3 μm to generate 20-bit TIF files according to the manufacturer’s protocols.

### Microarray data analysis

TIF files were extracted using Feature Extraction Software version 11 (Agilent Technologies, Santa Clara, CA, USA) and the GE1_1105_Oct12 protocol. The resulting raw data were analysed using DirectArray Software (OakLabs, Hennigsdorf, Germany). The signal distributions of the raw data were visualized using box plots to identify potential issues for individual samples.

Samples were quantile normalized and subjected to statistical analysis (PA *versus* GA) by applying Welch’s test and calculating log_2_ fold changes for each gene. *P* < 0·050 was considered statistically significant.

### Pathway analysis

Differentially expressed pathways were identified using the generally applicable gene set enrichment method of Luo and co-workers^14^ and the gage package in R (R Foundation for Statistical Computing, Vienna, Austria). The following immunologically relevant pathways are referred to in this analysis: Toll-like receptor signalling pathway; antigen processing and presentation; NOD-like receptor signalling pathway; haematopoietic cell lineage; natural killer cell-mediated cytotoxicity; tumour necrosis factor (TNF) signalling pathway and intestinal immune network for IgA production; complement and coagulation pathways; retinoic acid-inducible gene (RIG) I-like receptor signalling pathway; cytosolic DNA-sensing pathway; C-type lectin receptor signalling pathway; T cell receptor signalling pathway; Th1 and Th2 cell differentiation; Th17 cell differentiation; interleukin (IL) 17 signalling pathway; FcεRI signalling pathway; FcγR-mediated phagocytosis; and chemokine signalling pathway.

The significantly differentially expressed genes from the relevant pathways were visualized in a heatmap. For the colour representation, *Z*-scores were calculated for each line; *Z*-scores represent differences between a gene’s normalized signal for a sample and the gene’s mean signal for all samples divided by the standard deviation. The absolute *Z*-value represents the distance between a sample's gene signal and the gene’s mean signal of all samples in standard deviation units. A sample’s gene signal below the mean *Z*-value indicates a negative (blue) signal, whereas a signal above the mean *Z*-value indicates a positive (red) signal. Heatmap visualization was combined with hierarchical clustering, whereby samples with the most similar expression profiles—based on normalized expression values—were clustered together. Additionally, clusters were visualised as dendrograms, complementing the heatmaps.

## Results

**Fig. 2:**
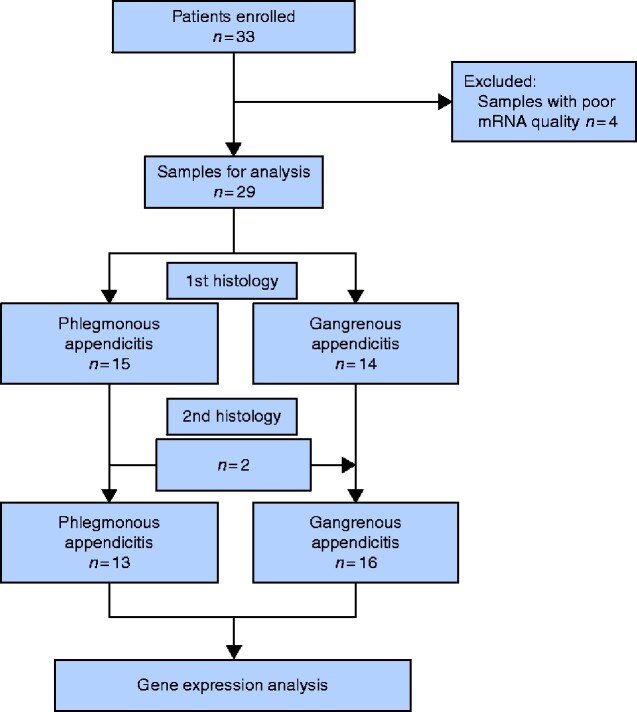
Patient flow diagram

Thirty-three otherwise healthy patients with sonographic suspicion of acute appendicitis were initially included. RNA quality control led to exclusion of four samples that had signs of degradation: three from patients with GA and one from a patient with PA. After primary routine histopathological examination, 15 specimens were classified as phlegmonous and 14 as gangrenous. After blind histopathological re-evaluation by a specialized paediatric pathologist, two samples from patients with a primary diagnosis of PA were classified as gangrenous inflammation. The PA group eventually consisted of 13 samples and the GA group of 16 (*Fig. 2*). No statistically significant differences were found with regard to age, sex distribution, and time from onset of symptoms to blood sample collection (*Table 1*).

**Table 1:** Distribution of age, sex, and duration of symptoms before sample collection

	**Total** **(*n* = 29)**	**Phlegmonous** **(*n* = 13)**	**Gangrenous** **(*n* = 16)**
Age (years)^*^	11.3(2.6)	11.5(2.7)	11.1(2.6)
Sex ratio (F : M)	14 : 15	4 : 9	10 : 6
Duration of symptoms before sample collection (h)^*^	28.3(16.9)	24.0(13.3)	32.1(19.4)

^*^Values are mean(s.d.).

### Gene expression analysis

Box plots of raw gene expression signals of the samples finally included and classified showed a similar distribution, with no obviously problematic samples (*Fig. S1*).

From a total of 56 666 protein-coding and non-coding RNAs analysed, 3594 (6.3 per cent) were significantly differentially expressed between the groups (*P* < 0·050). The distribution of expression patterns (log_2_ fold change *versus* –log (*P*)) with respect to significant upregulation and downregulation of genes is illustrated in a volcano plot (*Fig. S2*).

### Expression patterns within immunological pathways

The following immunological pathways were significantly differentially expressed: Toll and lmg signalling pathway, antigen processing and presentation, NOD-like receptor signalling pathway, haematopoietic cell lineage, natural killer cell-mediated cytotoxicity, TNF signalling pathway and intestinal immune network for IgA production, complement and coagulation pathways, RIG-I-like receptor signalling pathway, Cytosolic DNA-sensing pathway, C-type lectin receptor signalling pathway, T cell receptor signalling pathway, Th1 and Th2 cell differentiation, Th17 cell differentiation, IL-17 signalling pathway, FcεRI signalling pathway, FcγR-mediated phagocytosis, and chemokine signalling pathway. *Tables 2* and *3* show the significantly differentially regulated mRNAs. Information about the specific functions of the genes is provided in *Table S1*. Gene overexpression in the PA group was characterized by lymphoid cell lineage-directed patterns, specifically those involved in T cell/B cell interactions. In contrast, the expression pattern in the GA group was primarily assigned to an antibacterial function.

**Table 2:** Significantly differentially expressed genes with immunological relevance: relative gene overexpression in patients with phlegmonous appendicitis

Gene	Mean signal PA	Mean signal GA	***P*** ^†^
*MHC class II*	142	65	0·043
*HLA-F*	2395	1585	0·024
*HLA-DOB*	185	119	0·017
*CD40L* ^*^	444	250	0·003
*CD2*	715	304	0·027
*CD3*	224	121	0·021
*CD24*	144	87	0·021
*CD23*	582	276	0·010
*CD72*	47	27	0·023
*NIK*	150	83	0·029
*TRAF1*	147	93	0·042
*CD79* ^*^	4384	3148	0·004
*Pol-III*	66	42	0·037
*Interleukin 23*	74	30	0·027
*TCR β variable 2*	19	7	0·012
*TCR β variable 3-1*	7	3	0·019
*TCR β variable 4-2*	45	29	0·013
*TCR β variable 5-5* ^*^	7	4	0·002
*TCR β variable 5-6* ^*^	11	5	< 0·001
*TCR β variable 6*	55	27	0·013
*TCR β variable 6-5*	31	11	0·024
*TCR β variable 6-6*	23	9	0·015
*TCR β variable 6-8* ^*^	35	13	0·003
*TCR β variable 7-4* ^*^	17	7	0·008
*TCR β variable 7-7* ^*^	7	2	< 0·001
*TCR β variable 11-1* ^*^	13	6	0·002
*TCR β variable 11-3* ^*^	6	2	0·003
*TCR β variable 14*	23	9	0·045
*TCR β variable 15*	6	2	0·019
*TCR β variable 18*	17	8	0·022
*TCR β variable 29-1*	51	23	0·036
*TCR a variable 1-2*	19	10	0·016
*TCR α variable 2*	10	5	0·021
*TCR α variable 8-6*	15	8	0·042
*TCR α variable 10*	6	4	0·026
*TCR α variable 12-1* ^*^	7	3	0·007
*TCR α variable 12-2*	19	11	0·032
*TCR α variable 16*	8	3	0·018
*TCR α variable 17*	17	10	0·044
*TCR α variable 19* ^*^	23	10	0·004
*TCR α variable 22*	19	10	0·042
*TCR α variable 23*	17	8	0·021
*TCR α variable 24*	9	5	0·019
*TCR α variable 26-1*	8	6	0·048
*TCR α variable 35* ^*^	7	3	0·006
*TCR α variable 38-2*	25	15	0·028
*TCR α variable 41* ^*^	18	11	0·003

^*^Gene expression within the top 100 differentially expressed genes (based on *P* values). PA, phlegmonous appendicitis; GA, gangrenous appendicitis.

^†^Welch’s test.

**Table 3:** Significantly differentially expressed genes with immunological relevance: relative gene overexpression in patients with gangrenous appendicitis

Gene	Mean signal PA	Mean signal GA	***P*** ^†^
*CD11b*	325	409	0·029
*CD16b*	1048	1678	0·032
*CD35*	437	658	0·022
*CD55*	1121	1540	0·032
*CD36*	2159	3046	0·023
*TLR5*	754	1134	0·011
*TLR9*	116	134	0·022
*NLRP3*	135	188	0·034
*NLRC4*	100	148	0·026
*NLRP6*	139	167	0·011
*Cathelicidin*	29	37	0·050
*CD64*	65	127	0·022
*C9*	68	87	0·032
*SOCS3*	1217	2366	0·010
*IRAK4*	260	327	0·030
*Tpl2 kinase*	188	324	0·037
*IL-17A*	196	251	0·048
*LCN2*	162	276	0·023

PA, phlegmonous appendicitis; GA, gangrenous appendicitis.

^†^Welch’s test.

**Fig. 3:**
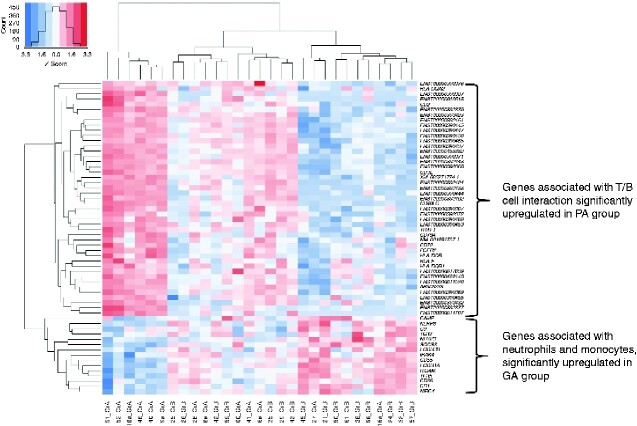
Heat map visualizing cluster formation of samples with similar immunological expression patterns Samples from patients with phlegmonous appendicitis (PA) (GrA) or gangrenous appendicitis (GA) (GrB). Signals above (red) and below (blue) the mean signal are shown.

Eleven of the significantly increased expressed genes in the PA group concerning α or β subunits of the T cell receptor were found within the top 100 expressed genes. In the GA group, two significantly increased mRNAs were found among the top 100: SOCS3 and neutrophilic CD64. Based on statistically significant *P* values within immunological pathways, a heat map for respective differentially expressed genes was created (*Fig. 3*).

## Discussion

Diagnostic methods provide the framework for the surgical options for treatment of acute appendicitis. The authors’ motivation beyond clarification of the pathophysiologies was to show what could be expected from gene expression data in the diagnosis of acute appendicitis in the future, especially with regard to one of the most affected age groups. The medium-term goal is to obtain the simplest possible molecular marker providing the most valid information possible for rapid therapeutic decision-making. Such a marker should be safe, objective (without need for interpretation), easy to determine and, in terms of prospective informative value, superior to other diagnostic tools such as imaging, clinical examination, standard laboratory parameters, and combinatorial scores. The latter are especially useful, but they represent complicated constructs with many variables, which have to be interpreted partially^15^.

Overall, 6.3 per cent of the genes analysed in this study were expressed significantly differently between the groups, demonstrating substantial differences among inflammatory entities. For complementary data analysis, the Kyoto Encyclopedia of Genes and Genomes was instrumentalized to map and link the disparately expressed genes to relevant molecular pathways. The results of this complex data analysis gave rise to the hypothesis that PA represents an immunological response to viral infections, whereas GA results in an expression pattern related to an antibacterial response.

Most theories generally designate luminal obstruction as the primary origin of acute appendicitis^2^. In contrast, however, studies have shown faecaliths in up to 27 per cent of all autopsies without inflammatory changes to the appendix, indicating that faecaliths are an incidental rather than a pathophysiologically causal finding in appendicitis specimens^2^. Nevertheless, new insights into the pathophysiology of acute inflammation of the vermiform appendix, which could replace the refuted theories, have been scarce.

In the present study, PA was characterized by lymphoid-directed gene expression patterns. Particularly, overexpression of α and β subunits of the T cell receptor, MHC class II and CD40L, T cell markers CD2 and CD3, and B-cell markers CD72 and CD79 in patients with PA suggested a specific immunological synapse between T and B cells^16–20^. That seems to be essential for the activation of B cells and the induction of an antiviral humoral response; B cells are reciprocally activated by cytokines for antibody production upon virus internalization, degradation, MHC class II-dependent antigen presentation, and recognition by Th2 cells via the T cell receptor–CD3 complex. Co-stimulation is provided by CD40/CD40L and CD2^19^^,^^20^^,^^16^^,^^17^. Overexpression of α and β subunits of the T cell receptor in the PA group only represented 11 per cent of the top 100 overexpressed genes. These results confirm previous findings regarding Th2 cell-dependent immunological mechanisms in patients with PA^6^^,^^7^. In contrast, patterns associated with myeloid lineage were found in patients with gangrenous inflammation. The monocyte and neutrophil markers CD11b, CD16b, and CD64 point to a primarily antibacterial defence^21^. In particular, the significant upregulation of the bacterial pattern recognition receptors toll-like receptor (TLR) 5 and TLR-9 supports this interpretation^22^^,^^23^. Consistent with the authors’ theory, low antiviral function is demonstrated by overexpression of suppressor of cytokine signalling (SOCS) 3, which inhibits antiviral cytokine signalling^24^. SOCS3 expression might even explain the relative overexpression of Th17 promoting interleukin 23 in patients with PA; SOCS3 is a potent negative regulator of interleukin 23 expression^25^. Thus, this seeming inconsistency might be explained by downregulation in GA rather than upregulation in PA.

Interestingly, the susceptibility of humans with the functional IL-17A rs2275913 polymorphism for appendicitis has been associated with advanced inflammation^13^. This finding correlates with the present observation of significantly enhanced IL-17A expression in patients with GA. Further to this, the pathway analysis especially contributes to the thesis of independent pathophysiologies. The specific functions of genes not mentioned here are tabulated in *Table S1*.

The study results are in accordance with previous immunological and infectiological investigations regarding acute appendicitis, and the authors’ hypothesis that PA and GA represent independent entities. Mean symptom duration and distribution of temporal patterns were comparable in the two groups. These findings are consistent with previous evidence of independent inflammatory courses in patients with PA and GA, without transition from one form to the other^3^^,^^4^.

Studies have shown that fetal development of the lymphatic tissue in the appendix is already characterized by persistence of T and B lymphocytes^26^. As a result, maturation of the intestinal immune system does not seem to solely depend on microbial and nutritional antigens. The presence of T and B lymphocytes may be linked to current theories about the basic function of the human vermiform appendix, as it has been suggested to provide a ‘safe house’ for commensal bacteria in the gut, especially during the course of viral diarrhoea. Subsequently, the commensal bacteria are protected by a biofilm while the composition of the associated lymphatic tissue ensures its antiviral function^27^. Indeed, several viruses, such as coxsackievirus, influenza virus, measles, cytomegalovirus, and adenovirus, have been associated with non-perforating inflammation of the appendix^28^^,^^29^.

Microarray-based comparative investigation for pathway analysis has been shown previously to be a highly effective tool^30^. However, despite numerous quality features such as its prospective design, the repeated histopathological assessment, strict quality control of the samples, and ambitious data analysis, the present study has essentially two limitations. Although expectations were met in terms of indicating substantial pathophysiological differences between PA and GA, a larger sample size could have revealed an even more comprehensive picture of the immunological pathways involved, as a large number of nearly statistically significant expression differences were found. Second, the inclusion of healthy patients as a control group could have provided more comprehensive insights.

The results presented here offer valuable information regarding the general description of differential gene expression and identification of specific yet independent immunological gene expression patterns in light of current research. They provide proof of concept for a comprehensive follow-up study with an adequate sample size calculation focusing on gene expression profiling, possibly coupled with high-performance algorithms, to allow successful development of a simple and reliable biomarker to distinguish inflammatory entities within acute appendicitis in childhood. The methods described here could therefore provide a fundamental framework for reliable diagnostics in acute appendicitis at a simple, low-invasive, low-risk, and cost-effective level.

## Funding

HRH Crown Princess Lovisa’s Foundation, Stockholm, Sweden.

## Supplementary Material

zraa045_Supplementary_Data
