# Supplementary material for: Use of gene expression profiling to identify candidate genes for pretherapeutic patient classification in acute appendicitis
Source: BJS Open. 2021 Jan 9;5(1):zraa045. doi: 10.1093/bjsopen/zraa045 (PMC7893459; doi:10.1093/bjsopen/zraa045)
Supplement: zraa045_Supplementary_Data [file zraa045_supplementary_data.zip › Suppl. TABLE 1 BJS Open.docx]

| **Gene** | **Function** |
| --- | --- |
| MHC class II | Antigene presentation^1^ |
| HLA-F | Expression on B- and T-cells, monocytes and NK-cells, antiviral function^2^ |
| HLA-DOB | Modulation of MHC class II antigene presentation on B cells^3^ |
| CD40L | Expression on activated T-cells, antiviral activation of B-cells^4^ |
| CD2 | Co-stimulatory molecule on T-cells for B-cell activation^5,6^ |
| CD3 | Part of TCR/CD3 complex for T- and B-cell activation^1,6^ |
| CD24 | Response modulation in activated B-cells^7,8^ |
| CD23 | Surface marker on B cells, CD40/CD40L-dependent expression^8,9^ |
| CD72 | Response modulation in activated B-cells^10^ |
| NIK | Regulator of TCR/CD3 pathway^11^ |
| TRAF1 | Enhances CD40 signals to B-cells^12^ |
| CD79 | BCR accessory molecule^13^ |
| Pol-III | Detection of viral dsDNA, induction of IFN-beta production^14^ |
| Interleukin 23 | Promotes formation of Th17 cells^15^ |
| TCR beta variable 2 | TCR subunit^16^ |
| TCR beta variable 3-1 | TCR subunit^16^ |
| TCR beta variable 4-2 | TCR subunit^16^ |
| TCR beta variable 5-5 | TCR subunit^16^ |
| TCR beta variable 5-6 | TCR subunit^16^ |
| TCR beta variable 6 | TCR subunit^16^ |
| TCR beta variable 6-5 | TCR subunit^16^ |
| TCR beta variable 6-6 | TCR subunit^16^ |
| TCR beta variable 6-8 | TCR subunit^16^ |
| TCR beta variable 7-4 | TCR subunit^16^ |
| TCR beta variable 7-7 | TCR subunit^16^ |
| TCR beta variable 11-1 | TCR subunit^16^ |
| TCR beta variable 11-3 | TCR subunit^16^ |
| TCR beta variable 14 | TCR subunit^16^ |
| TCR beta variable 15 | TCR subunit^16^ |
| TCR beta variable 18 | TCR subunit^16^ |
| TCR beta variable 29-1 | TCR subunit^16^ |
| TCR alpha variable 1-2 | TCR subunit^17^ |
| TCR alpha variable 2 | TCR subunit^17^ |
| TCR alpha variable 8-6 | TCR subunit^17^ |
| TCR alpha variable 10 | TCR subunit^17^ |
| TCR alpha variable 12-1 | TCR subunit^17^ |
| TCR alpha variable 12-2 | TCR subunit^17^ |
| TCR alpha variable 16 | TCR subunit^17^ |
| TCR alpha variable 17 | TCR subunit^17^ |
| TCR alpha variable 17 | TCR subunit^17^ |
| TCR alpha variable 19 | TCR subunit^17^ |
| TCR alpha variable 22 | TCR subunit^17^ |
| TCR alpha variable 23 | TCR subunit^17^ |
| TCR alpha variable 24 | TCR subunit^17^ |
| TCR alpha variable 26-1 | TCR subunit^17^ |
| TCR alpha variable 35 | TCR subunit^17^ |
| TCR alpha variable 38-2 | TCR subunit^17^ |
| TCR alpha variable 41 | TCR subunit^17^ |

| **Gene** | **Function** |
| --- | --- |
| CD11b | Adhesion molecule on neutrophils and monocytes/macrophages, correlation with activation^18^ |
| CD16B | Expression on neutrophils, induction of ROS production^19^ |
| CD35 | Complement receptor 1, binds immune complexes with C3b/C4b and mediates uptake by macrophages and neutrophils^20^ |
| CD55 | Expression on neutrophils and monocytes, protection of the cells against damage by complement C3b/C4b^21^ |
| CD36 | Surface marker on monocytes/macrophages, induces phagocytosis^22^ |
| TLR5 | Pattern recognition receptor for bacterial flagellin^23^ |
| TLR9 | Pattern recognition for bacterial and viral DNA^24^ |
| NLRP3 | Pattern recognition receptor for bacteria, viruses, fungi and parasites; inhibition by T-cells via CD40L^25^ |
| NLRC4 | Functional complex formation with NLRP^25^ |
| NLRP6 | Pattern recognition receptor, regulation of inflammation and host defense against microorganisms^26^ |
| Cathelicidin | Antimicrobial peptide expressed e.g. in neutrophils, disrupting cell membranes^27^ |
| CD64 | Monocyte/macrophage and neutrophil surface marker, correlation with cell activation^28^ |
| C9 | Member of the antimicrobial complement membrane attack complex, disruption of cell membranes^29^ |
| SOCS3 | Suppression of antiviral cytokine signaling^30^ |
| IRAK4 | Essential for TLR signaling^31^ |
| Tpl2 kinase | Regulation of inflammatory response in macrophages and neutrophils^32^ |
| IL-17A | Produced by Th17 cell subset, important for defense against bacterial and fungal infections^33^ |
| LCN2 | Antimicrobial defense mediator by binding bacterial siderophores. important for neutrophil function^34^ |

**Suppl. Table 1 Significantly differentially expressed genes: specific functions**

**a**

**b**

Functions of genes with a: relative gene overexpression in patients with phlegmonous appendicitis (PA), and b: relative gene overexpression in patients with gangrenous appendicitis (GA).

**References**

1 Mills DM, Cambier JC. B lymphocyte activation during cognate interactions with CD4+ T lymphocytes: Molecular dynamics and immunologic consequences. *Semin Immunol* 2003; **15**: 325–329.

2 Hò G-GT, Heinen FJ, Blasczyk R, Pich A, Bade-Doeding C. HLA-F Allele-Specific Peptide Restriction Represents an Exceptional Proteomic Footprint. *Int J Mol Sci* 2019; **20**: 5572.

3 Douek DC, Altmann DM. HLA-DO is an intracellular class II molecule with distinctive thymic expression. *Int Immunol* 1997; **9**: 355-364.

4 Whitmire JK, Slifka MK, Grewal IS, Flavell RA, Ahmed R. CD40 ligand-deficient mice generate a normal primary cytotoxic T-lymphocyte response but a defective humoral response to a viral infection. *J Virol* 1996; **70**: 8375–8381.

5 Noelle RJ, Snow EC. T helper cell-dependent B cell activation. *FASEB J* 1991; **5**: 2770-2776.

6 Clevers H, Alarcon B, Wileman T, Terhorst C. The T cell receptor/CD3 complex: A dynamic protein ensemble. *Annu Rev Immunol* 1988; **6**: 629–662.

7 Kay R, Rosten PM, Humphries RK. CD24, a signal transducer modulating B cell activation responses, is a very short peptide with a glycosyl phosphatidylinositol membrane anchor. *J Immunol* 1991; **147**: 1412-1416.

8 Kaminski DA, Wei C, Qian Y, Rosenberg AF, Sanz I. Advances in human B cell phenotypic profiling. *Front Immunol* 2012; **3**: 302.

9 Sukumar S, Conrad DH, Szakal AK, Tew JG. Differential T Cell-Mediated Regulation of CD23 (FcεRII) in B Cells and Follicular Dendritic Cells. *J Immunol* 2006; **176**: 4811-4817.

10 Adachi T, Wakabayashi C, Nakayama T, Yakura H, Tsubata T. CD72 Negatively Regulates Signaling Through the Antigen Receptor of B Cells. *J Immunol* 2000; **164**: 1223–1229.

11 Matsumoto M, Yamada T, Yoshinaga SK, Boone T, Horan T, Fujita S *et al.* Essential Role of NF-κB-Inducing Kinase in T Cell Activation Through the TCR/CD3 Pathway. *J Immunol* 2002; **169**: 1151–1158.

12 Xie P, Kraus ZJ, Stunz LL, Bishop GA. Roles of TRAF molecules in B lymphocyte function. *Cytokine Growth Factor Rev* 2008; **19**: 199–207.

13 Chu PG, Arber DA. CD79: A review. *Appl Immunohistochem Mol Morphol* 2001; **9:** 97-106.

14 Chiu Y-H, MacMillan JB, Chen ZJ. RNA Polymerase III Detects Cytosolic DNA and Induces Type I Interferons through the RIG-I Pathway. *Cell* 2009; **138**: 576–591.

15 Chen Z, Laurence A, Kanno Y, Pacher-Zavisin M, Zhu B-M, Tato C *et al.* Selective regulatory function of Socs3 in the formation of IL-17-secreting T cells. *Proc Natl Acad Sci* 2006; **103**: 8137–8142.

16 Born W, Yague J, Palmer E, Kappler J, Marrack P. Rearrangement of T-cell receptor β-chain genes during T-cell development. *Proc Natl Acad Sci U S A* 1985; **82**: 2925–2929.

17 Klein MH, Concannon P, Everett M, Kim LD, Hunkapiller T, Hood L. Diversity and structure of human T-cell receptor alpha-chain variable region genes. *Proc Natl Acad Sci U S A* 1987; **84**: 6884-6888.

18 Muller Kobold AC, Tulleken JE, Zijlstra JG, Sluiter W, Hermans J, Kallenberg CGM *et al.* Leukocyte activation in sepsis: Correlations with disease state and mortality. *Intensive Care Med*. 2000; **26**: 883–892.

19 Wright HL, Moots RJ, Bucknall RC, Edwards SW. Neutrophil function in inflammation and inflammatory diseases. *Rheumatol* 2010; **49:** 1618–1631.

20 Dunkelberger JR, Song WC. Complement and its role in innate and adaptive immune responses. *Cell Res* 2010; **20**: 34-50.

21 Berger M, Medof ME. Increased expression of complement decay-accelerating factor during activation of human neutrophils. *J Clin Invest* 1987; **79**: 214–220.

22 Fadok VA, Warner ML, Bratton DL, Henson PM. CD36 is required for phagocytosis of apoptotic cells by human macrophages that use either a phosphatidylserine receptor or the vitronectin receptor (alpha v beta 3). *J Immunol* 1998; **61**: 6250-6257.

23 Yoon S Il, Kurnasov O, Natarajan V, Hong M, Gudkov A V., Osterman AL *et al.* Structural basis of TLR5-flagellin recognition and signaling. *Science* 2012; **335**: 859-864.

24 El Kebir D, József L, Pan W, Wang L, Filep JG. Bacterial DNA Activates Endothelial Cells and Promotes Neutrophil Adherence through TLR9 Signaling. *J Immunol* 2009; **182**: 4386–4394.

25 Man SM, Hopkins LJ, Nugent E, Cox S, Glück IM, Tourlomousis P *et al.* Inflammasome activation causes dual recruitment of NLRC4 and NLRP3 to the same macromolecular complex. *Proc Natl Acad Sci U S A* 2014; **111:** 7403-7408.

26 Levy M, Shapiro H, Thaiss CA, Elinav E. NLRP6: A Multifaceted Innate Immune Sensor. *Trends Immunol* 2017; **38**: 248-260.

27 Zanetti M. Cathelicidins, multifunctional peptides of the innate immunity. *J Leukoc Biol* 2004; **75**: 39-48.

28 Hoffmann JJML. Neutrophil CD64: A diagnostic marker for infection and sepsis. *Clin Chem Lab Med* 2009; **47**: 903-916.

29 Dudkina N V., Spicer BA, Reboul CF, Conroy PJ, Lukoyanova N, Elmlund H *et al.* Structure of the poly-C9 component of the complement membrane attack complex. *Nat Commun* 2016; **7**: 10588.

30 Song MM, Shuai K. The suppressor of cytokine signaling (SOCS) 1 and SOCS3 but not SOCS2 proteins inhibit interferon-mediated antiviral and antiproliferative activities. *J Biol Chem* 1998; **273**: 35056-35062.

31 Tae WK, Staschke K, Bulek K, Yao J, Peters K, Oh KH *et al.* A critical role for IRAK4 kinase activity in Toll-like receptor-mediated innate immunity. *J Exp Med* 2007; **204**: 1025–1036.

32 Xu D, Matsumoto ML, McKenzie BS, Zarrin AA. TPL2 kinase action and control of inflammation. *Pharmacol Res* 2018; **129**: 188–193.

33 Iwakura Y, Ishigame H, Saijo S, Nakae S. Functional Specialization of Interleukin-17 Family Members. *Immunity* 2011; **34**: 149–162.

34 Liu Z, Petersen R, Devireddy L. Impaired Neutrophil Function in 24p3 Null Mice Contributes to Enhanced Susceptibility to Bacterial Infections. *J Immunol* 2013; **190**: 4692–4706.
