# Supplementary figures and images for: Use of gene expression profiling to identify candidate genes for pretherapeutic patient classification in acute appendicitis
Source: BJS Open. 2021 Jan 9;5(1):zraa045. doi: 10.1093/bjsopen/zraa045 (PMC7893459; doi:10.1093/bjsopen/zraa045)

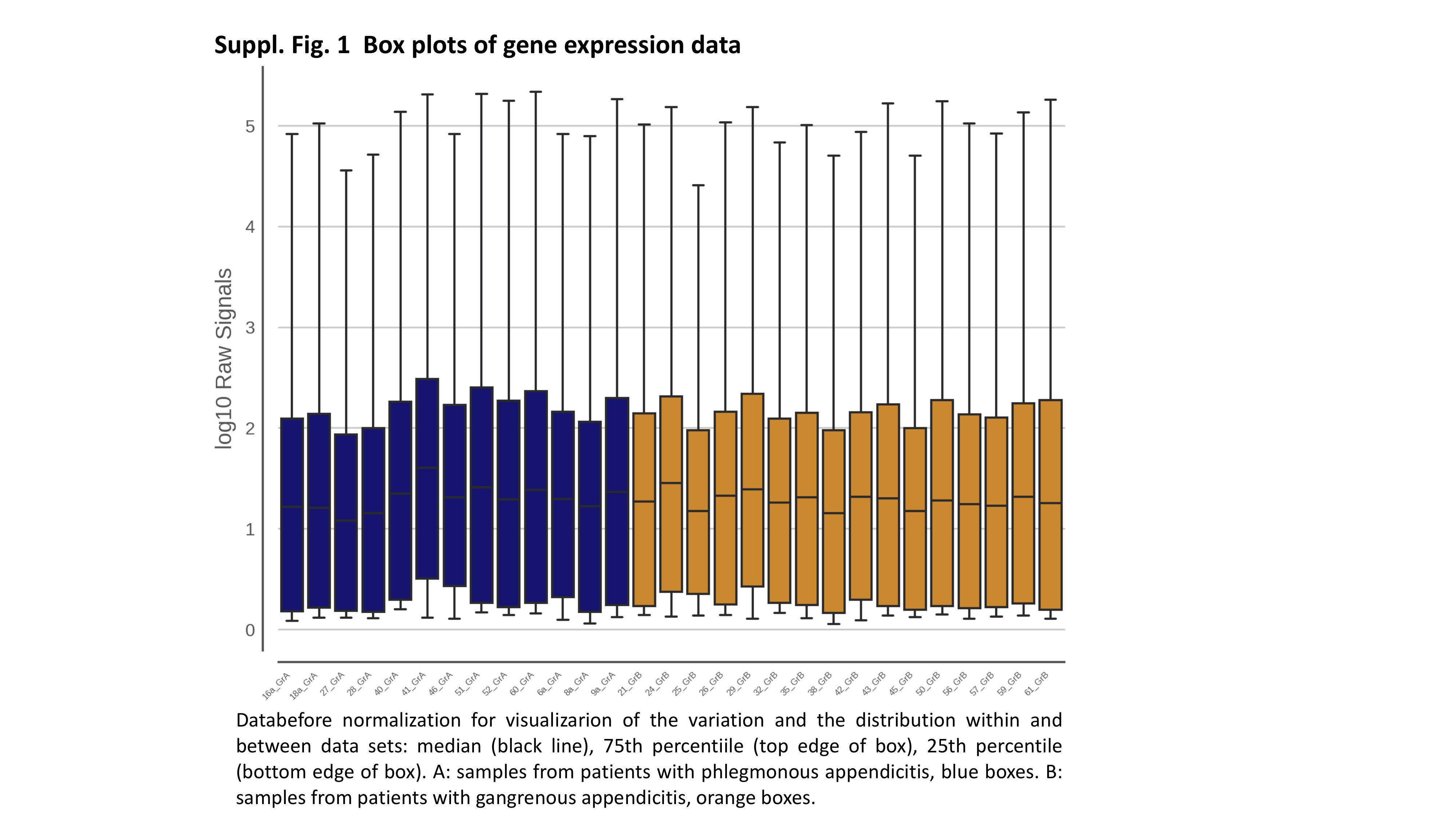

Supplement: zraa045_Supplementary_Data [file zraa045_supplementary_data.zip › Suppl. Fig. 1 Br J Surg.jpg]

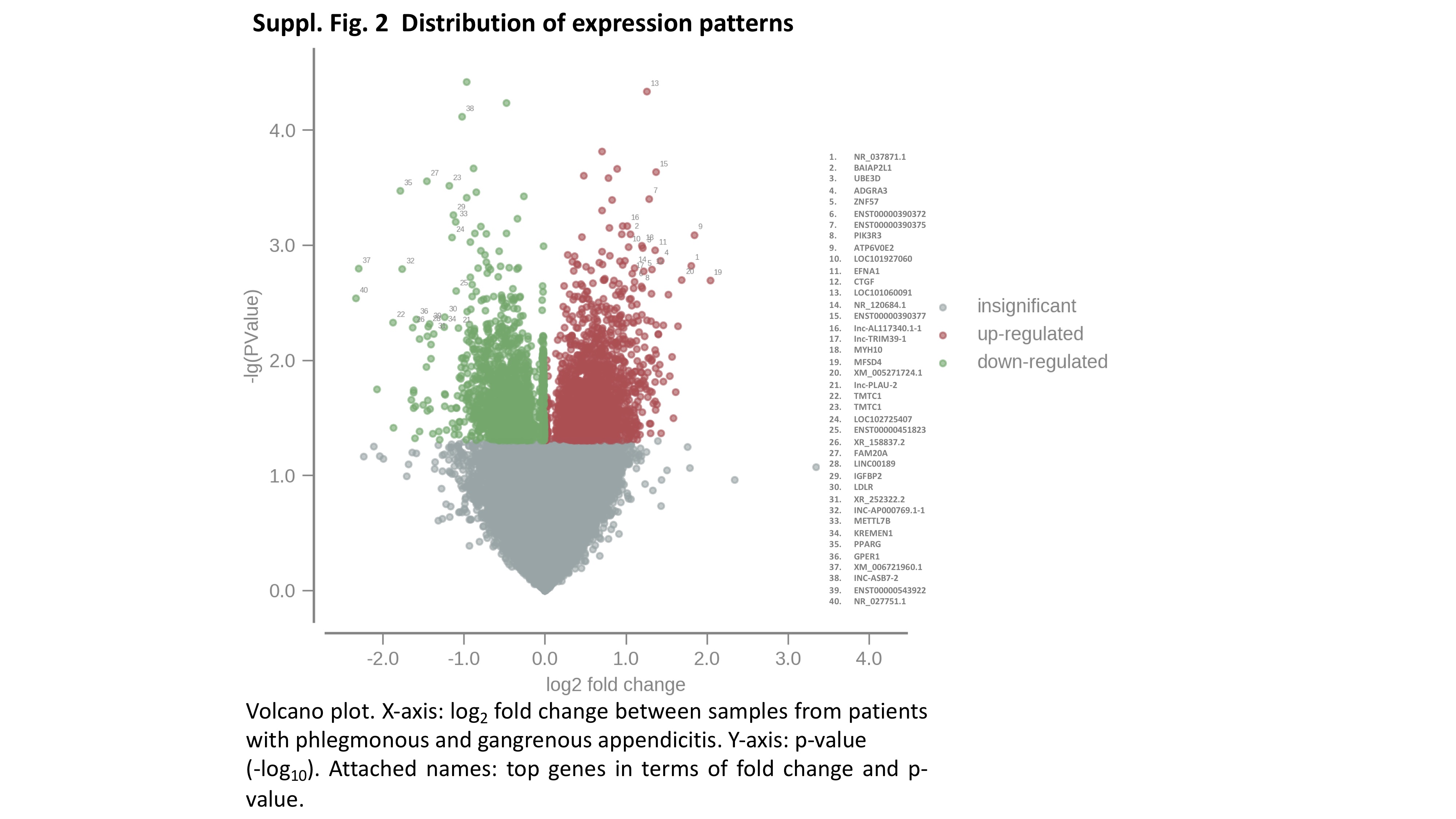

Supplement: zraa045_Supplementary_Data [file zraa045_supplementary_data.zip › Suppl. Fig. 2 Br J Surg.jpg]
